# Revealing the Critical Regulators of Cell Identity in the Mouse Cell Atlas

**DOI:** 10.1016/j.celrep.2018.10.045

**Published:** 2018-11-06

**Authors:** Shengbao Suo, Qian Zhu, Assieh Saadatpour, Lijiang Fei, Guoji Guo, Guo-Cheng Yuan

**Affiliations:** 1 Department of Biostatistics and Computational Biology, Dana-Farber Cancer Institute and Harvard T.H. Chan School of Public Health, Boston, MA 02215, USA; 2 Center for Stem Cell and Regenerative Medicine, Zhejiang University School of Medicine, Hangzhou 310058, China; 3 These authors contributed equally; 4 Lead Contact

## Abstract

Recent progress in single-cell technologies has enabled the identification of all major cell types in mouse. However, for most cell types, the regulatory mechanism underlying their identity remains poorly understood. By computational analysis of the recently published mouse cell atlas data, we have identified 202 regulons whose activities are highly variable across different cell types, and more importantly, predicted a small set of essential regulators for each major cell type in mouse. Systematic validation by automated literature and data mining provides strong additional support for our predictions. Thus, these predictions serve as a valuable resource that would be useful for the broad biological community. Finally, we have built a user-friendly, interactive web portal to enable users to navigate this mouse cell network atlas.

## In Brief

Suo et al. create a mouse cell network atlas by computational analysis of previously published single-cell RNA-seq data. They predict essential regulators for all major cell types in mouse and develop an interactive web portal for query and visualization.

## Graphical Abstract

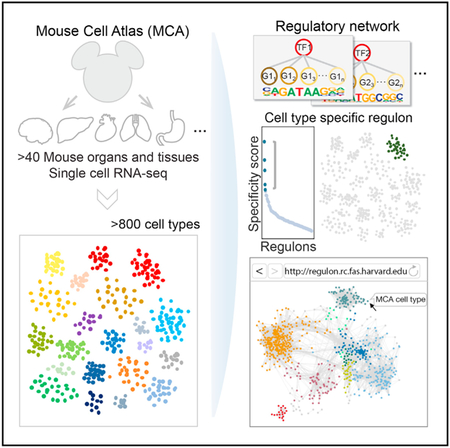

## INTRODUCTION

A multi-cellular organism contains diverse cell types; each has its own functions and morphology. A fundamental goal in biology is to characterize the entire cell-type atlas in human and model organisms. With the rapid development of single-cell technologies, great strides have been made in the past few years (Svensson et al., 2018). Multiple groups have made tremendous progresses in mapping cell atlases in complex organs (such as mouse brain and immune system) (Rosenberg et al., 2018; Saunders et al., 2018; Stubbington et al., 2017; Zeisel et al., 2018), early embryos (such as in *C. elegans* and zebrafish) (Cao et al., 2017; Wagner et al., 2018), or even entire adult animals (such as *Schmidtea mediterranea* and mouse) (The Tabula Muris Consortium et al., 2018; Fincher et al., 2018; Han et al., 2018; Plass et al., 2018). International collaborative efforts are underway to map out the cell atlas in human (Regev et al., 2017).

How do cells maintain their identity? While it is clear the maintenance of cell identity involves the coordinated action of many regulators, transcription factors (TFs) have been long recognized to play a central role. In several cases, the activity of a small number of key TFs, also known as the master regulators, are essential for cell identity maintenance: depletion of these regulators cause significant alteration of cell identity, while forced expression of these regulators can effectively reprogram cells to a different cell type (Han et al., 2012; Ieda et al., 2010; Riddell et al., 2014; Takahashi and Yamanaka, 2006). However, for most cell types, the underlying gene regulatory circuitry is incompletely understood. With the increasing diversity of gene expression programs being identified through single-cell analysis, an urgent need is to understand how these programs are established during development, and to identify the key regulators responsible for such processes.

Systematic approaches for mapping gene regulatory networks (GRNs) have been well established. The most direct approach is through genome-wide occupancy analysis, using experimental assays such as chromatin immunoprecipitation sequencing (ChIP-seq), chromatin accessibility, or long-range chromatin interaction assays (ENCODE Project Consortium, 2012). However, this approach is not scalable to a large number of cell types, and its application is often limited by the number of cells that can be obtained in vivo. An alternative, more generalizable approach is to computationally reconstruct GRNs based on single-cell gene expression data (Fiers et al., 2018), followed by more focused experimental validations. In this study, we took this latter approach to build a comprehensive mouse cell network atlas.

To this end, we took advantage of the recently mapped mouse cell atlas (MCA) derived from comprehensive single-cell transcriptomic analysis (Han et al., 2018), and combined with a computational algorithm to construct GRNs from single-cell transcriptomic data. Our analysis indicates that most cell types have distinct regulatory network structure and identifies regulators that are critical for cell identity. In addition, we provide an interactive web-based portal for exploring the mouse cell network atlas.

## RESULTS

### Reconstructing Gene Regulatory Networks Using the MCA

**Figure 1.**
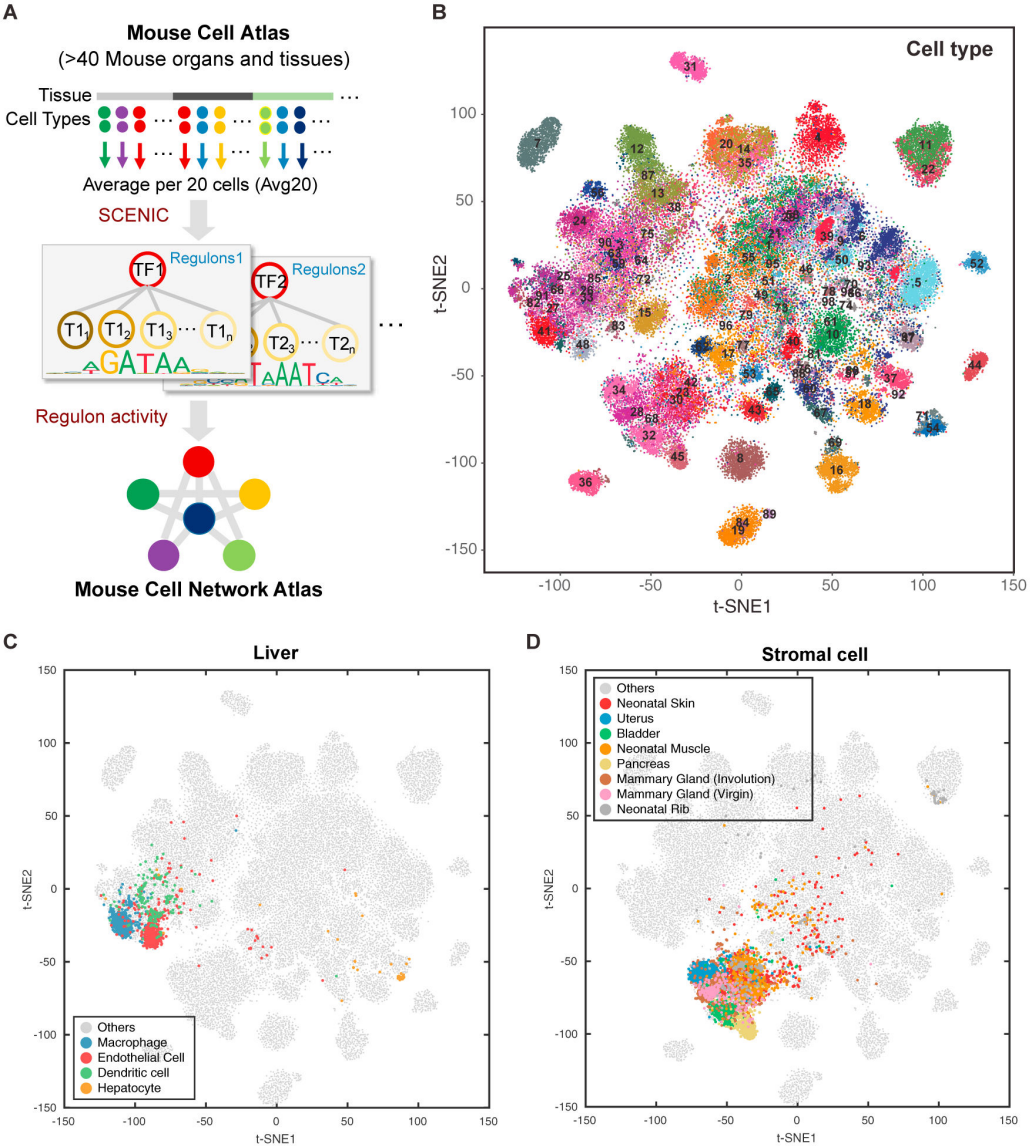
Mapping Mouse Cell Network Atlas with Regulon Activity (A) Schematic overview of the computational approach in this study. A modified SCENIC pipeline is used to infer cell-type-specific gene regulatory networks. (B–D) t-SNE map for all sampled single cells (~61 k) based on regulon activity scores (RAS), each cell is color-coded based on major cell-type assignment. (B) All sampled cells (~61 k) are highlighted. (C) Liver cells are highlighted. (D) Stromal cells are highlighted. See also Figures S1 and S2 and Tables S1, S2, and S3.

To comprehensively reconstruct the gene regulatory networks for all major cell types, we applied the SCENIC pipeline (Aibar et al., 2017) to analyze the MCA data. In brief, SCENIC links *cis*-regulatory sequence information together with single-cell RNA sequencing (RNA-seq) data. SCENIC contains three main steps, including co-expression analysis, target gene motif enrichment analysis, and regulon activity evaluation. The main outcomes contain a list of regulons (each representing a TF along with a set of co-expressed and motif significantly enriched target genes), and the regulon activity scores (RAS) for each cell (Figure 1A). To improve computational efficiency and robustness, we modified the original pipeline to analyze pooled data instead. Specifically, we divided the entire cell population into small groups with similar cell states. This was achieved by random, non-overlapping sampling from the same tissue and cell type. Each group contains 20 cells. We applied SCENIC to infer regulons based on the group-averaged gene expression profiles, and the RAS scores were calculated at the single-cell level as in the original study (Aibar et al., 2017). By testing its performance on several representative tissues, we found that our modified approach separated cell types more effectively compared to the original implementation (Figure S1; see STAR Methods for details).

We focused on a representative, well-annotated subset of MCA data (Han et al., 2018), containing 61,637 cells sampled from 43 tissues. Previous analysis has identified 98 main cell types (Han et al., 2018). By applying the modified SCENIC approach described above, we identified 202 significant regulons containing 8,461 genes (Table S1).

The size of each regulon varies from 10–2,502 genes, with a median size of 73 genes. Strikingly, different cell types are well-separated using the RAS-based distance (Figures 1B and S2A; Table S2). Even within a single tissue, different cell types can be separated based on the regulon activities (Figures 1C and S2B). For example, each of four main cell types identified in liver occupies a distinct territory in the t-Distributed Stochastic Neighbor Embedding (tSNE) plot (Figure 1C).

One question of interest is whether cells of the same type may have different regulatory circuitries across tissues. Such differences would be relevant for investigating cell-environment interactions (Figures 1D and S2C). To address this question, we focused on stromal cells, which can be found in a wide variety of tissues, providing support, structural and anchoring functions. The behavior of stromal cells is well known to be highly plastic, a necessary property for supporting a diverse range of tissue development (Lee et al., 2006). Indeed, we found that the stromal cells from different tissues tend to have distinct regulon activities (Figure 1D). While stromal cells are clustered together from the global view of t-SNE map, closer examination suggests the subpopulations from different tissues—such as uterus, mammary gland, bladder, and pancreas—are well separated. Similar refined structure can be found in various other cell types, such as T cells and epithelial cells (Figure S2C). Taken together, regulon-based activity score in different cell types provides a new avenue to investigate the potential regulatory mechanism in inter- and intra-cell type variations. Our analysis indicates that GRN differences are primarily driven by cell type differences but further modulated by tissue environment differences.

### Comparative Analysis Identified Essential Regulators for the Maintenance of Cell Identity

**Figure 2.**
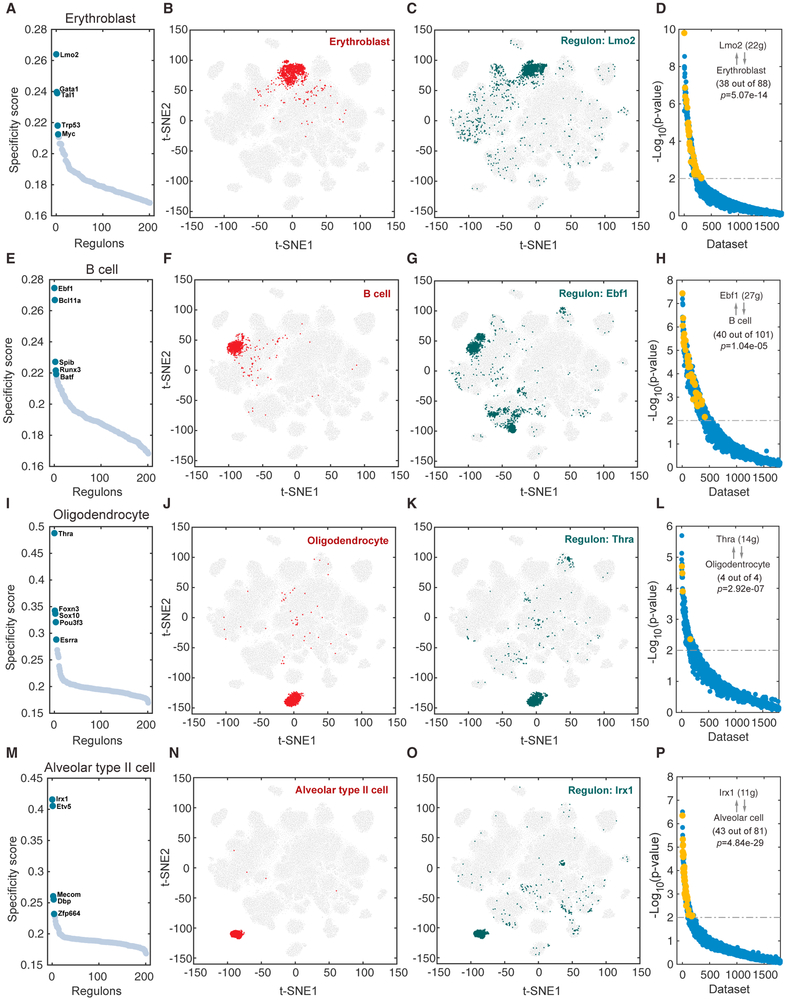
Cell-Type-Specific Regulon Activity Analysis (A–D) Erythroblast. (A) Rank for regulons in erythroblast cell based on regulon specificity score (RSS). (B) Erythroblast cells are highlighted in the t-SNE map (red dots). (C) Binarized regulon activity scores (RAS) (do Z score normalization across all samples, and set 2.5 as cutoff to convert to 0 and 1) for top regulon *Lmo2* on t-SNE map (dark green dots). (D) SEEK co-expression result for target genes of top regulon *Lmo2* in different GEO datasets. The x axis represents different datasets, and the y axis represents the co-expression significance of target genes in each dataset. Erythroblast related datasets with significant correlation (p value < 0.01) are highlighted by yellow dots. (E–H) Same as (A)–(D) but for B cells. (I–L) Same as (A)–(D) but for oligodendrocytes. (M–P) Same as (A)–(D) but for alveolar type II cells. See also Figure S2 and Table S4.

Our comprehensive network analysis provides an opportunity to systematically identify critical regulators for cell identity. For each regulon, we evaluated its activities associated with each of the 98 major cell types (Table S3), and defined a regulon specificity score (RSS) based on Jensen-Shannon divergence (Cabili et al., 2011) Table S3; see STAR Methods). We then selected the regulons with highest RSS values and further examined their functional properties. To test whether this approach is effective, we started with the erythroblast because its core gene regulatory network has been well characterized (Orkin and Zon, 2008). Our network analysis identified *Lmo2*, *Gata1*, and *Tal1* (also known as SCL), as the most specific regulons associated with erythroblast (Figure 2A). tSNE plot provides additional support that the activities of these regulons are highly specific to erythroblast (Figures 2B and 2C). Of note, all three factors are well-known master regulators for erythrocytes (Welch et al., 2004; Wilson et al., 2010; Wu et al., 2014). Another well-characterized cell type is the B cell. Our network analysis identified *Ebf1* and *Bcl11a* as the most specific regulons (Figures 2E–2G). Both factors are well known to be essential regulators for maintaining B cell identity (Liu et al., 2003; Nechanitzky et al., 2013).

The success of our approach in recapitulating critical regulators for well-characterized cell types motivated us to repeat the analysis for all other 96 cell types. For each cell type, we identified a small number of regulons with exceptionally high specificity scores (Figures 2, S2D, and S2E). To systematically evaluate the accuracy of these predictions, we used two complementary approaches: SEEK (Zhu et al., 2015) and CoCiter (Qiao et al., 2013), based on mining the pubic datasets and literatures, respectively. First, SEEK analysis was done to select datasets in which the TF and its target genes within a regulon are co-expressed. We queried the titles for public mouse datasets for enrichment of cell-type-specific terms, with the assumption that functionally related genes tend to be co-expressed in the corresponding cell types. Second, CoCiter analysis was done to identify enriched co-occurrence of a gene and cell type term pair in publication abstracts, with the assumption that functionally related genes and terms should frequently appear together in the literature.

To test if the above two data-mining approaches are useful validation strategies, we applied each approach to test the regulators identified for erythroblast and B cells. For erythroblast, we applied SEEK analysis to search for GEO datasets in which the genes in regulon *Lmo2* are significantly co-expressed. Among the more than 2,000 datasets examined by SEEK, the erythroblast related datasets are highly ranked (Figure 2D; Table S4, Fisher’s exact test, p = 5.07e–14; see STAR Methods). Similarly, we applied CoCiter analysis to search for publications where genes in regulon *Lmo2* co-occur with the term “erythroblast/erythrocyte.” Again, the result is highly specific (co-citation impact [CI] = 8.41, permutation p < 0.001, see STAR Methods). Similarly, for B cells, the top ranked datasets in which *Ebf1* and its target genes are co-expressed tend to be associated with B cells (Figure 2H, p = 1.04e–05), and these genes tend to co-occur in studies related to B cells (CI = 9.38, permutation p < 0.005).

The above analysis indicates that both SEEK and CoCiter analyses are useful for validating predicted essential regulators of cell identity. Therefore, we applied both approaches to evaluate the relevance of predicted essential regulators for other cell types, most of which are incompletely characterized. For example, oligodendrocytes are a type of neuroglia whose main functions are to provide support and insulation to axons in the CNS. While various factors have been implicated to play a role in oligodendrocyte development (Zuchero and Barres, 2013), the most important regulators remain unknown. Our network analysis found that regulon *Thra* shows the highest specificity score (Figures 2I–2K), suggesting this may be one of the most essential regulators for oligodendrocyte identity. As additional support, SEEK analysis indicated that the genes in regulon *Thra* are significantly co-expressed in oligodendrocyte related datasets (Figure 2L, p = 2.92e–07). For the potential target genes of *Thra*, CoCiter analysis shows that there are 204 papers (CI = 7.68, permutation p < 0.005) mentioned both the genes of regulon *Thra* and “oligodendrocyte” in their abstracts.

Approximately 15% of lung cells belong to alveolar type II (AT2), which has the important functions of synthesizing and secreting surfactant (Mason, 2006). Our GRN analysis indicates that regulon *Irx1* shows the highest specificity score in these cells (Figures 2M–2O), indicating this TF may be an essential regulator for AT2 cell identity. As additional support, SEEK analysis shows the genes in regulon *Irx1* significantly co-express in alveolar related datasets (Figure 2P, p = 4.84e–29). Literature mining finds that the genes in this regulon are associated with “alveolar” (CI = 8.02, permutation p value < 0.001). Taken together, these results strongly indicate that our predicted cell-type-specific regulators are functionally relevant.

### Regulons Are Organized into Combinatorial Modules

**Figure 3.**
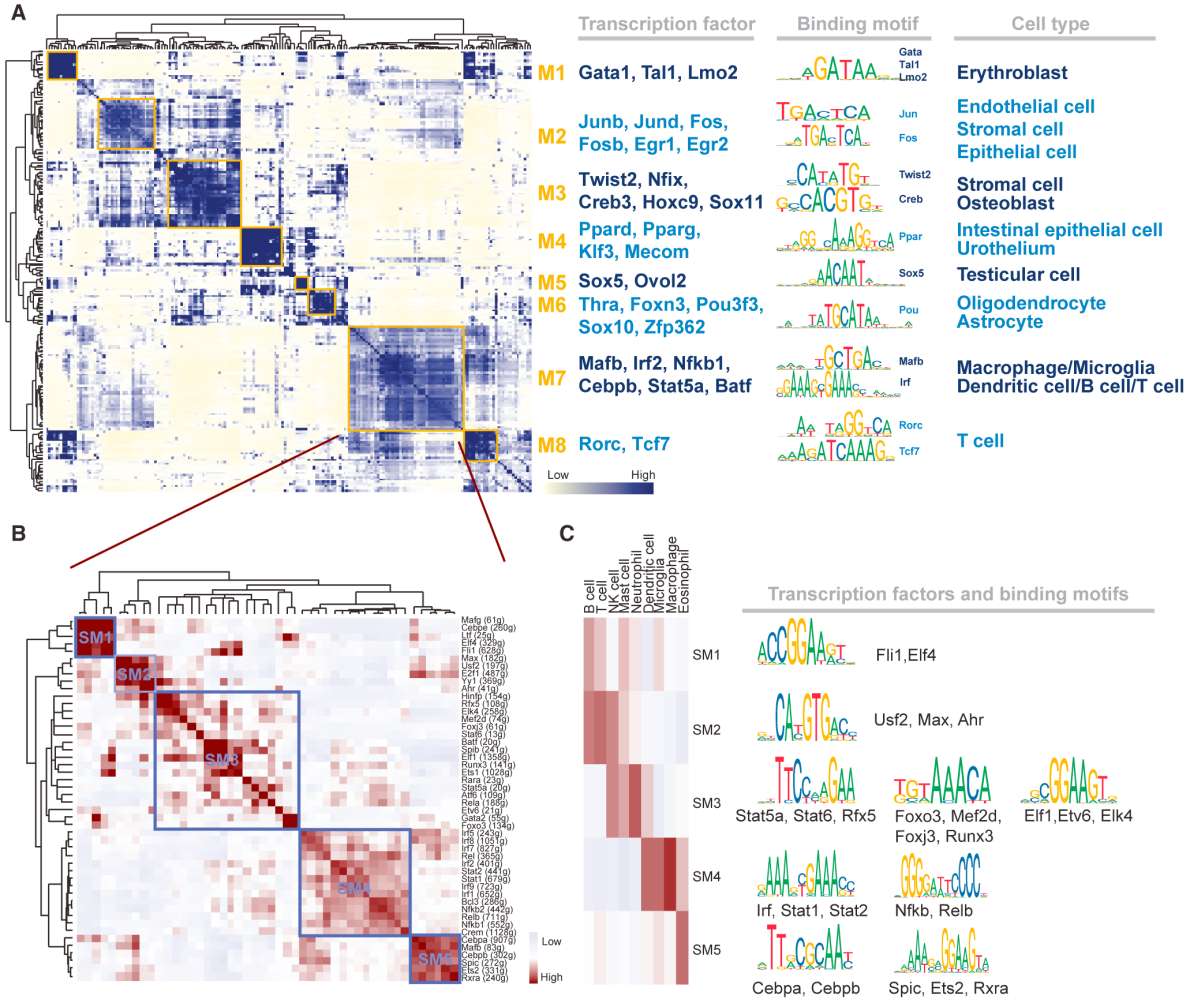
Identification of Combinatorial Regulon Modules (A) Identified regulon modules based on regulon connection specificity index (CSI) matrix, along with representative transcription factors, corresponding binding motifs, and associated cell types. (B) Zoomed-in view of module M7 identifies sub-module structures. (C) Different sub-modules in M7 are associated with distinct immune cell types and regulon activities. See also Figure S3.

Transcription factors often work in combination to coordinate gene expression levels. To systematically characterize the combinatorial patterns, we compared the atlas-wide similarity of RAS scores of every regulon pair based on the Connection Specificity Index (CSI) (Fuxman Bass et al., 2013) (see STAR Methods). Strikingly, these 202 regulons are organized into 8 major modules (Figures 3A and S3A). For each module, we identified several representative regulators and cell types through their average activity scores (Figure S3B). When mapping the average activity score of each module onto tSNE map, we found that each module occupies distinct region and all highlighted regions show complementary patterns (Figure S3C). Module M1 contains regulators *Gata1*, *Tal1*, and *Lmo2*, which are essential regulators for the erythroblast. M2 contains regulators that are associated with epithelial proliferation and endothelial apoptosis, such as *Jun* and *Fos* (Okada et al., 2004; Wang et al., 1999). M3 contains a mixture of stroma-specific factors, such as *Twist2* and *Nfix* (Galván et al., 2015; Grabowska et al., 2014), as well as regulators for bone formation, such as *Creb* family members (Murakami et al., 2009). Several M4 regulators, such as *Ppard* (Shi et al., 2017) and *Klf3* (Kaga et al., 2017), are associated with epithelial cells. Module M5 contains regulators that are specifically activated in testicular cells, such as Sox5 (Kiselak et al., 2010) and *Ovol2* (Chizaki et al., 2012). Regulons in M6 are highly associated with the nervous system, such as oligodendrocytes and astrocytes. The activity of M7 including *Mafb*, *Irf2*, and *Nfkb1* is specifically high in different immune cell types (Valledor et al., 1998), such as macrophages, microglia, dendritic cells, B cells, and T cells. Module M8 is also related to immune cells but it is very specifically enriched in one subtype of T cells that are derived from the thymus tissue (cluster 8) (Figures 1B and S3C). A closer examination of this cell type indicates that the regulon *Rorc* is specifically activated in this subtype. It is known that RORγt (encoded by *Rorc*) is essential for T cell maturation in the thymus and could suppress conventional effector responses such as proliferation and cytokine production (Sun et al., 2000; Yui and Rothenberg, 2014), which indicates this subtype of T cells might be in a “naive” state. In contrast, the T cell subtypes that are associated with M7 have distinct regulon activities and reside in other tissues. For example, T cells from clusters 3 and 15 are mainly from mammary gland tissues. In these subtypes, the regulon *Batf* (contained in M7) has the highest specificity score. Because *Batf* has been identified to play a fundamental role in regulating the differentiation of effector of CD8^+^ T cells (Kurachi et al., 2014), these two T cell subtypes are likely to be in an “activated” state.

We next focused on the largest module M7, which contains 48 regulons. This module is strongly associated with immune cell types. This is perhaps not surprising, considering the complexity of the mammalian immune system. Consistent with this observation, the target genes in these regulons are enriched for immune related functions, such as “defense response” and “immune system response.” A closer look reveals that M7 can be further divided into 5 smaller sub-modules (SM1–SM5) (Figure 3B). Interestingly, each smaller module is specifically associated with distinct immune cell types (Figure 3C). For example, SM3 is associated with neutrophils, and the involved regulons including *Stat5a* (Fiévez et al., 2007), *Foxo3* (Osswald et al., 2018), and *Elf1* (Bjerregaard et al., 2003) are associated with neutrophil regulation. SM4 is mainly related to macrophages; correspondingly, many well-known macrophage-related regulators are predicted by our network analysis, such as *Irf* family genes and *Nfkb* genes (Valledor et al., 1998). Regulons in SM5 are highly activated in eosinophil cells, and it has been shown that C/EBP genes are required for eosinophil lineage commitment and maturation (Nerlov et al., 1998). These analyses indicate a hierarchical organization of regulatory modules that work together in fine-tuning cellular states.

### Mapping the MCA Network Using Cell-Type-Specific Regulatory Activity

**Figure 4.**
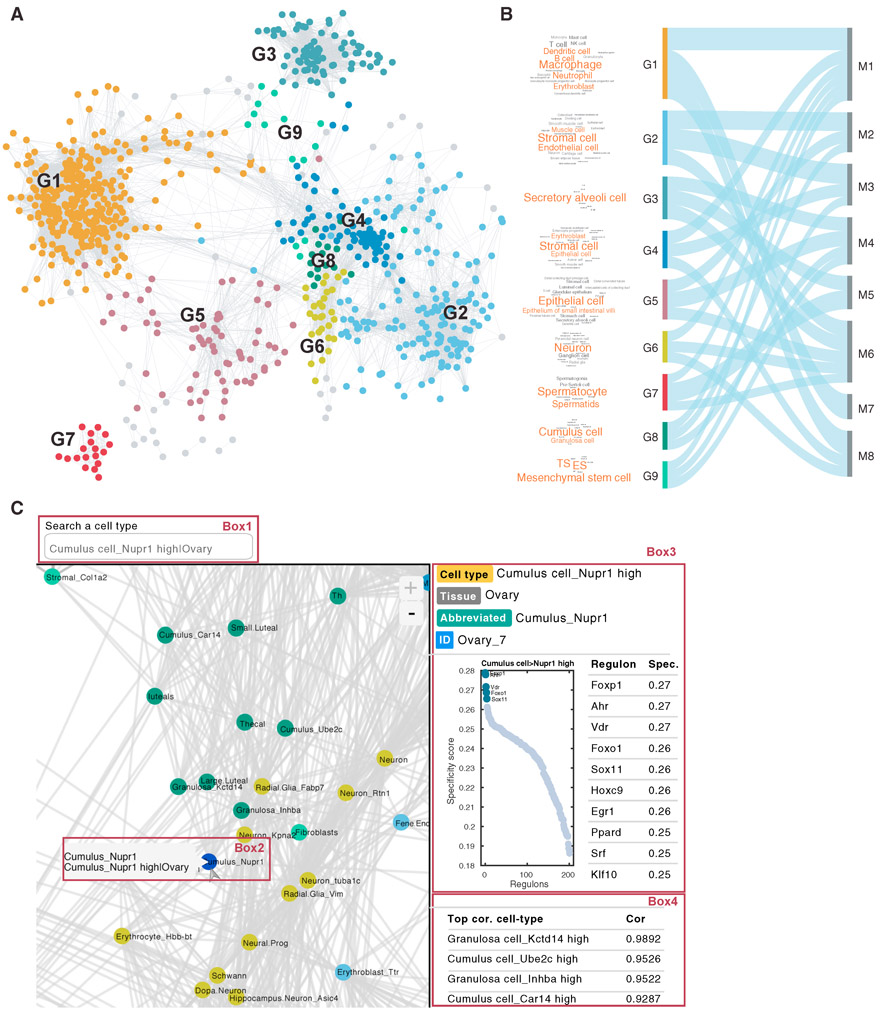
A Summary View of the Mouse Cell Network Atlas (A) Relatedness network for the 818 cell types based on similarity of regulon activities. Each group represents a set of highly related cell types. (B) Sankey plot shows relationship between cell-type groups G1–G9 and regulon modules M1–M8, the thematic cell type composition within each cluster is indicated by the corresponding wordcloud plot. (C) A representative screenshot of the web portal obtained by querying “cumulus cells.” See also Figure S4 and Tables S2 and S3.

The full MCA dataset contains over 800 cell types (Table S2). At this resolution, many cell types share similar gene expression patterns and their biological functions are likely to be less distinct. Our complete network analysis estimated the RAS and RSS (Table S3) for all these cell types. We found that related cell types share similar overall network structure. Such relationship can be well-represented by a highly modularized graph, where each edge connects a pair of related cell types whose overall regulon activities are similar (Spearman correlation coefficient>0.8) (Figure 4A). The global network structure is similar to that of the 98 major cell types (Figure S4). This graph can be further divided into 21 groups (G1–G21) by using the Markov clustering algorithm (MCL) (see STAR Methods), with functional-related cell types being clustered together. Nine of these groups (G1–G9) contain more than 10 related cell types (Figures 4A and 4B). For example, G1 contains a number of blood-related cell types, whereas cell types in G2 perform various supporting functions. The Sankey plot (Figure 4B) summarizes the relationship between cell types and their top associated regulon modules. For example, M1, M7, and M8, which are immune cell-type-related regulon modules (Figure 3A), are highly enriched in G1, whereas modules M2 and M3 (stromal cell-related modules) (Figure 3A) are highly enriched in G2.

### A Web-Based Resource for Interrogating Mouse Cell Network Atlas

We created a web-based portal to enable users to easily navigate this predicted mouse cell network atlas (http://regulon.rc.fas.harvard.edu/). The web interface provides both regulon-centric and cell-type centric views. The regulon-centric view represents the relationship between the 202 regulons; each edge connects a pair of regulons whose cell-type-specific activity scores are highly correlated. Similarly, the cell-type centric view represents the relationship between the cell types; each edge connects a pair of cell types that share similar regulon activity patterns. The user can choose between the 98 major cell types (that are analyzed here) and 818 cell types (from the whole MCA) versions.

As an example, cumulus cells are a special cell type in the ovary whose function is not well characterized. To find information about this cell type, a user could simply enter “cumulus cell” in the search box to search for related cell types (Figure 4C, Box 1). This will lead to a menu containing related cell types identified by the MCA, one of these cell types is encoded as “Cumulus cell_Nupr1 high” (Figure 4C, Box 2), as *Nupr1* is a distinct marker for this cell type. Selecting this cell type would generate two main outputs. The first output is a list of regulons ranked by the degree of RSS. The most specific regulons are *Foxp1*, *Arh*, and *Vdr*, although a number of additional regulons, such as *Foxo1*, have similar specificity (Figure 4C, Box 3). The second output is a list of cell types with similar regulatory networks. Not surprisingly, its neighboring cell types include other cumulus cell subtypes identified by MCA. Of interest, granulosa cells, which are also called cumulus granulosa cells depending on location within the ovarian follicle, are also identified as its neighbors (Figure 4C, Box 4). Thus, biomedical investigators can identify putative regulons that are likely important during mouse ovary and oocyte development. The web portal also provides a zoom function, which enables users to interactively explore the regulon and cell-type network structures at any desired resolution. In addition, the raw data are also downloadable from the web portal to support further investigation.

## DISCUSSION

Our knowledge of cellular heterogeneity has exploded in the past few years. In comparison, for most of the cell types identified so far, we lack mechanistic understanding how their characteristic gene expression programs are established and maintained. Neither do we understand the developmental and functional relationship between different cell types. Such information is not just of fundamental biological interest, but also can guide developing novel cell reprogramming strategies with clinical implications. Building upon the recently mapped MCA (Han et al., 2018), we have comprehensively constructed the GRNs for all major cell types in mouse through computational analysis. An important consequence is the predictions of critical regulators for each cell type. While most predictions remain hypotheses, they have provided a guide for future experimental investigation. As such, we have created a valuable resource for the broad biologist community.

## STAR★METHODS

### LEAD CONTACT

Further information and requests for resources should be directed to and will be fulfilled by the Lead Contact, Guo-Cheng Yuan (gcyuan@jimmy.harvard.edu).

### METHOD DETAILS

#### Datasets

The MCA dataset and the corresponding cell type annotation were downloaded from https://figshare.com/articles/MCA_DGE_Data/5435866. It contains two parts: (1) the subsampled ~61K single cell dateset, which includes 98 major cell types and covers 43 tissues and organs; (2) the whole MCA dataset, which contains over 250K single cells after removing the low-quality cells mapped to 818 cell types.

#### Inference of regulons and their activity

A number of computational methods have been developed to predict gene regulatory networks from single-cell gene expression data (Fiers et al., 2018). Here we used a modified version of the SCENIC (Single-Cell rEgulatory Network Inference) approach (Aibar et al., 2017; Davie et al., 2018) for constructing GRNs from single-cell RNaseq data (Han et al., 2018). Briefly, SCENIC contains three steps: (1)identify co-expression modules between TF and the potential target genes; (2) for each co-expression module, infer direct target genes based on those potential targets for which the motif of the corresponding TF is significantly enriched. Each regulon is then defined as a TF and its direct target genes; (3) the RAS in each single cell is calculated through the area under the recovery curve.

The original implementation of SCENIC is not scalable to large datasets and its results can be significantly affected by sequencing depth. To improve the scalability or robustness, we modified its implimentation by pooling data from every 20 cells randomly selected within each cell type and tissue and then applying SCENIC to the average gene expression profile of the pooled data. This simple modification (referred to as Avg20) effectively increases the data quality as well as reduces the computational burden.

We compared the performance of our modified approach with the original version of SCENIC by analyzing the MCA data from three representative tissues: bladder, kidney and bone marrow. For performance evaluation, we calculated the Silhouette value, which is a commonly used quantitative metric for clustering consistency. Specifically, the silhouette value *S_i_*, for the *i*th cell is defined as *S_i_* = (*b_i_*-*a_i_*)/max(*b_i_*,*a_i_*), where *a_i_* is the average distance between the *i*th single cell and the rest from the same cell type, and *b_i_* is the minimum average distance between the *i*th cell and any cell from a different cell type. A high silhouette value indicates a high degree of separation among cell types; therefore, it provides a quantitative metric for functional relevance. For each tissue, the Avg20 approach was repeated three times to estimate the variability due to random sampling, and t test was used to evaluate whether the performance of Avg20 approach is better than that of using all single cells. The results are shown in Figure S1. The consistency between three replicates was evaluated by the following approaches. First, the overlap among the TFs of regulons was evaluated by Fisher’s exact test. Second, for each Avg20 replicate, we calculated the pairwise distance of single cells in each tissue based on their RAS and then calculated Pearson correlation coefficient (PCC) to evaluate the agreement of different Avg20 replicates.

#### Quantifying cell-type specificity score

To quantify the cell-type specificity of a regulon, we adapted an entropy-based strategy that was previously used for gene expression data analysis (Cabili et al., 2011). First, we use a vector $P^{R}=(p_{1}^{R},\;\ldots ,p_{n}^{R})$ to represent the distribution of RAS in the cell population (*n* is the total number of cells). Here, the RAS are normalized so that $\sum_{i=1}^{n}p_{i}^{R}=1$. Then, we use a vector $P^{C}=(p_{1}^{C},\;\ldots ,p_{n}^{C})$ to indicate whether a cell belongs to a specific cell-type $\left(p_{i}^{C}=1\right)$ or not $\left(p_{i}^{C}=0\right)$. This vector is also normalized so that $\sum_{i=1}^{n}p_{i}^{C}=1$. Next, we evaluate the Jensen-Shannon Divergence (JSD), which is a commonly used metric for quantifying the difference between two probability distributions, defined as

$$JSD(P^{R},P^{C})=H\left(\frac{P^{R}+P^{C}}{2}\right)-\frac{H(P^{R})+H(P^{C})}{2}$$

where *H*(*P*) = -Σ *p_i_* log(*p_i_*) represents the Shannon entropy of a probability distribution P. The range of JSD values is between 0 and 1, where 0 means identical distribution and 1 means extreme difference. Finally, the regulon specificity score (RSS) is defined by converting JSD to a similarity score:

$$RSS(R,C)=1-\sqrt{JSD(P^{R},P^{C})}$$

For each cell type, the essential regulators are predicted as those associated with the highest cell-type specific scores.

#### Functional validation

We apply the following two methods to validate whether the predicted regulons are functional related to their associated cell types: (1) SEEK analysis (Zhu et al., 2015), and (2) CoCiter analysis (Qiao et al., 2013). First, SEEK (http://seek.princeton.edu/modSeek/mouse/) is a tool that provides the gene co-expression search function for over ~2000 mouse datasets from the Gene Expression Omnibus (GEO). We used the mouse version of SEEK to evaluate whether the genes in a regulon are co-expressed, and if so whether the datasets supporting the co-expression are associated with an interested cell type. If genes are significantly co-expressed in many datasets related to a certain cell type, it could be inferred that the function of this regulon is highly related to this cell type. Taking the erythroblast for example, we input gene list of regulon *Lmo2* to SEEK web server and search for erythroblast related keywords (such as ‘erythroblast’, ‘hematopoietic’, etc.) from the complete dataset-list ranked by query-coexpression score. Then we choose a p < 0.01 cutoff to select significant datasets and finally use Fisher’s exact test to evaluate whether the selected datasets are significantly enriched in the top ranks. Second, the CoCiter (Qiao et al., 2013) is a text mining approach against the up-to-date Medical Literature Analysis and Retrieval System Online (MEDLINE) literature database to evaluate the co-citation impact (CI, log-transformed paper count) between a gene list and a term. To assess significance of co-citation, a Monte Carlo approach is used to evaluate random expectations by randomly selecting 1000 gene sets with the same size as input gene list and then a permutation *p* value is calculated as the number of times that CI_random_ > CI_true_ divided by 1000. Here we used the function “gene-term” in CoCiter (use default parameters but set organism as mouse, http://www.picb.ac.cn/hanlab/cociter) to check whether the genes in a regulon are significantly co-cited with a certain cell type in literatures.

#### Regulon module analysis

Regulon modules were identified based on the Connection Specificity Index (CSI) (Fuxman Bass et al., 2013), which is a context-dependent measure for identifying specific associating partners. The evaluation of CSI involves two steps. First, the Pearson correlation coefficient (PCC) of activity scores is evaluated for each pair of regulons. Next, for a fixed pair of regulons, A and B, the corresponding CSI is defined as the fraction of regulons whose PCC with A and B is lower than the PCC between A and B.

Hierarchical clustering with Euclidean distance was performed based on CSI matrix to identify different regulon modules. We also used CSI > 0.7 as a cutoff to build the regulon association network to investigate the relationship of different regulons. The result was visualized by Cytoscape (Shannon et al., 2003). We used the same strategy to identify submodules within M7. For each regulon module, its activity score associated with a cell type is defined as the average of the activity scores of its regulon members in all cells within this cell type. Then the top ranked cell types are identified for each module.

#### Quantifying cell type relationship

Using the gene regulatory network analysis as a guide, we quantified the relationship between different cell-types based on the similarity of the overall regulon activities, which is quantified by the Spearman correlation coefficient. The results were represented as a network, where a pair of cell types were connected if the Spearman correlation coefficient is greater than 0.8. Again, the result was visualized by using Cytoscape. Groups of related cell-types were identified by using the Markov Clustering Algorithm (MCL) (van Dongen and Abreu-Goodger, 2012), as implemented in the ClusterMaker application in Cytoscape. We used the default setting except setting the inflation parameter as 2.

#### Web service

We created an interactive, web-based portal to explore the network atlas in this study (URL: http://regulon.rc.fas.harvard.edu). This interactive website is constructed with some of latest technologies including JavaScript libraries jQuery 3.3, Bootstrap 4, and Leaflet 1.3. Together these libraries provide efficient client-side search, zooming functions for the large cell type network. The site is hosted on an Apache web server running the Apache Tomcat which provides the necessary back-end support for the web server. Users can zoom-in on a part of network, mouse-over, click on a cell type in the network, and browse information about the associated regulons and other most similar cell types. The website also provides a complete, downloadable list of pairwise regulon-cell type associations.

### QUANTIFICATION AND STATISTICAL ANALYSIS

Details of the statistical tests used in this study are described briefly in the main text and more in-depth in the subsections above. They are also summarized below:

(1) To evaluate the consistency of identified regulons in three Avg20 replicates in each of bladder, kidney and bone marrow tissue, we counted the number of overlapped regulon TFs between different replicates and applied the one-sided Fisher’s exact test to evaluate statistical significance.
(2) t test was used evaluate whether the performance of Avg20 approach is better (p < 0.05) than that of using all single cells in each of bladder, kidney and bone marrow tissue based on silhouette value.
(3) When applying SEEK to test whether the genes in regulons are co-expressed in certain cell type, we chose correlation significant datasets (p < 0.01) and then used one-sided Fisher’s exact test to evaluate whether datasets related to interested cell type are significantly enriched (p < 0.01) in the top ranks.
(4) In CoCiter analysis, a permutation p value was introduced. It randomly selected 1000 gene sets with the same size of tested regulon and the p value was calculated as the number of times that co-citation impact of “random” larger than “true” divided by 1000.

### ADDITIONAL RESOURCES

We created an interactive, web-based portal for community to explore the network atlas in this study. URL: http://regulon.rc.fas.harvard.edu.

## Supplementary Material

1

2

3

4

5

6

## Figures and Tables

**Table:** KEY RESOURCES TABLE

REAGENT or RESOURCE	SOURCE	IDENTIFIER
Deposited Data		
Mouse Cell Atlas (MCA)	Han et al., 2018	https://figshare.com/articles/MCA_DGE_Data/5435866 (or GEO: GSE108097)
Software and Algorithms		
SCENIC	Aibar et al., 2017	https://github.com/aertslab/SCENIC
SEEK	Zhu etal., 2015	http://seek.princeton.edu/modSeek/mouse/
CoCiter (v2.3)	Qiao et al., 2013	http://www.picb.ac.cn/hanlab/cociter
tSNE (MATLAB implementation)	van der Maaten and Hinton, 2008	https://lvdmaaten.github.io/tsne/
R (v3.4.3)	The R Foundation	https://www.r-project.org
MATLAB (R2017a)	MathWorks	https://www.mathworks.com/products/matlab.html
Cytoscape (v3.5.1)	Shannon et al., 2003	https://cytoscape.org
Cluster 3.0 (Linux/Unix)	de Hoon et al., 2004	http://bonsai.hgc.jp/~mdehoon/software/cluster/software.htm
TreeView	Saldanha, 2004	http://jtreeview.sourceforge.net
Connection Specificity Index (CSI)	Fuxman Bass et al., 2013	http://lovelace.cs.umn.edu/similarity_index/guide.php
googleVis	R package for Google Visualization API	https://github.com/mages/googleVis
Other		
MCA Regulatory Network	This paper	http://regulon.rc.fas.harvard.edu

## Highlights

- Computational reconstruction of gene regulatory networks for major cell types in mouse
- Cell-type-specific regulons are organized into eight combinatorial modules
- Prediction of a small set of essential regulators for each cell type
- An interactive web portal for navigating predicted mouse cell network atlas
