# Supplementary material for: Revealing the Critical Regulators of Cell Identity in the Mouse Cell Atlas
Source: Cell Rep. Author manuscript; Available in PMC 2018 Dec 5. (PMC6281296; doi:10.1016/j.celrep.2018.10.045)
Supplement: 1 [file NIHMS1512149-supplement-1.pdf]

Cell Reports, Volume *25*

Supplemental Information

Revealing the Critical Regulators of Cell Identity in the Mouse Cell Atlas

Shengbao Suo, Qian Zhu, Assieh Saadatpour, Lijiang Fei, Guoji Guo, and Guo-Cheng Yuan

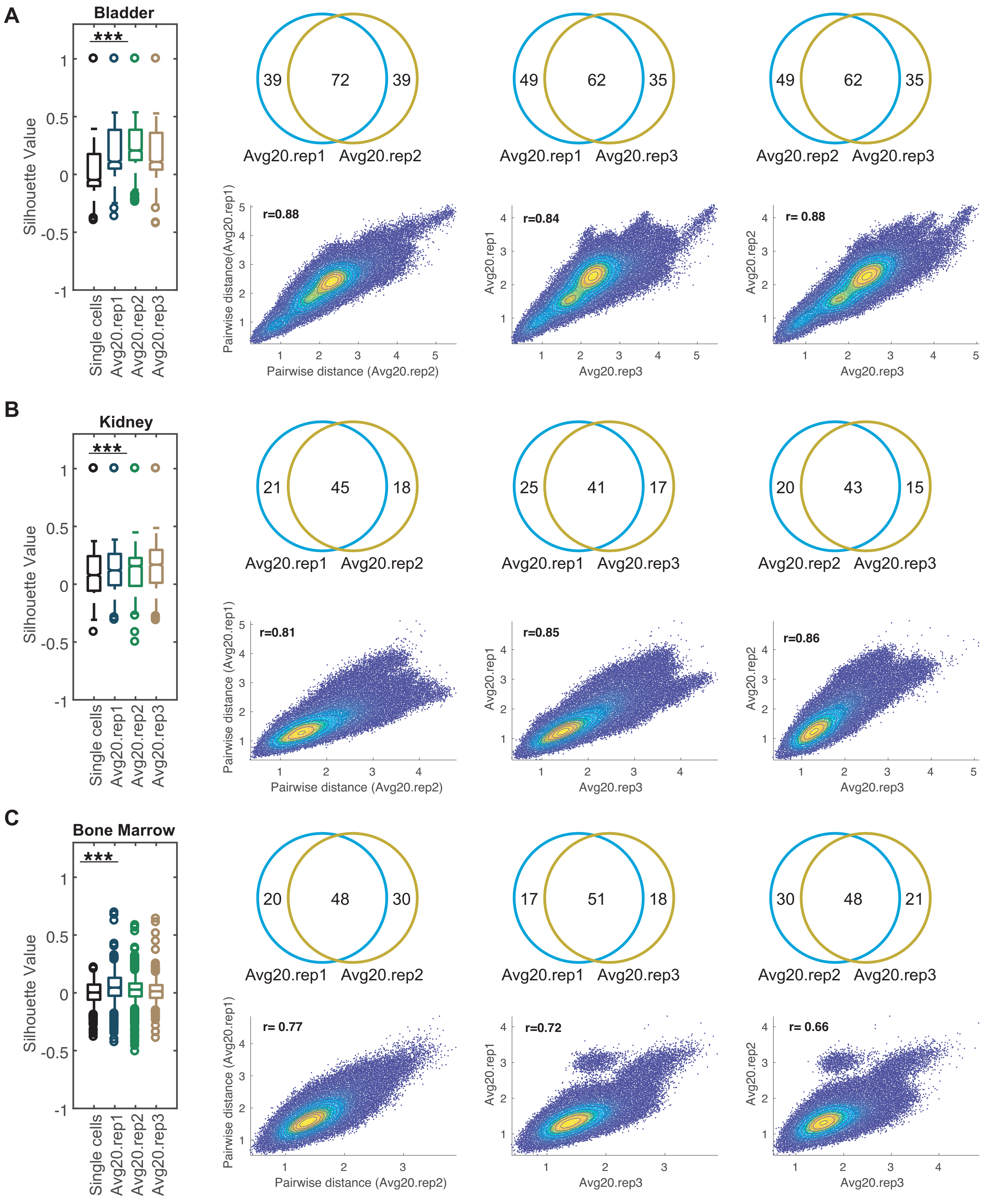

Figure S1. Comparison of our Avg20 approach with the original SCENIC implementation. Related to Figure 1.
(A) Bladder; (B) Kidney; (C) Bone marrow. For each tissue, the comparison is done in several ways. Left panels: comparison of RAS-based silhouette values (p<0.0001, t-test). The Avg20 approach was repeated three times (n=3). Top right panels: overlap of detected regulons between different Avg20 replicates (p<1e-22, Fisher's exaxt test). Bottom right panels: correlation between different Avg20 replicates based on pairwise distance of all single cells in each tissue.

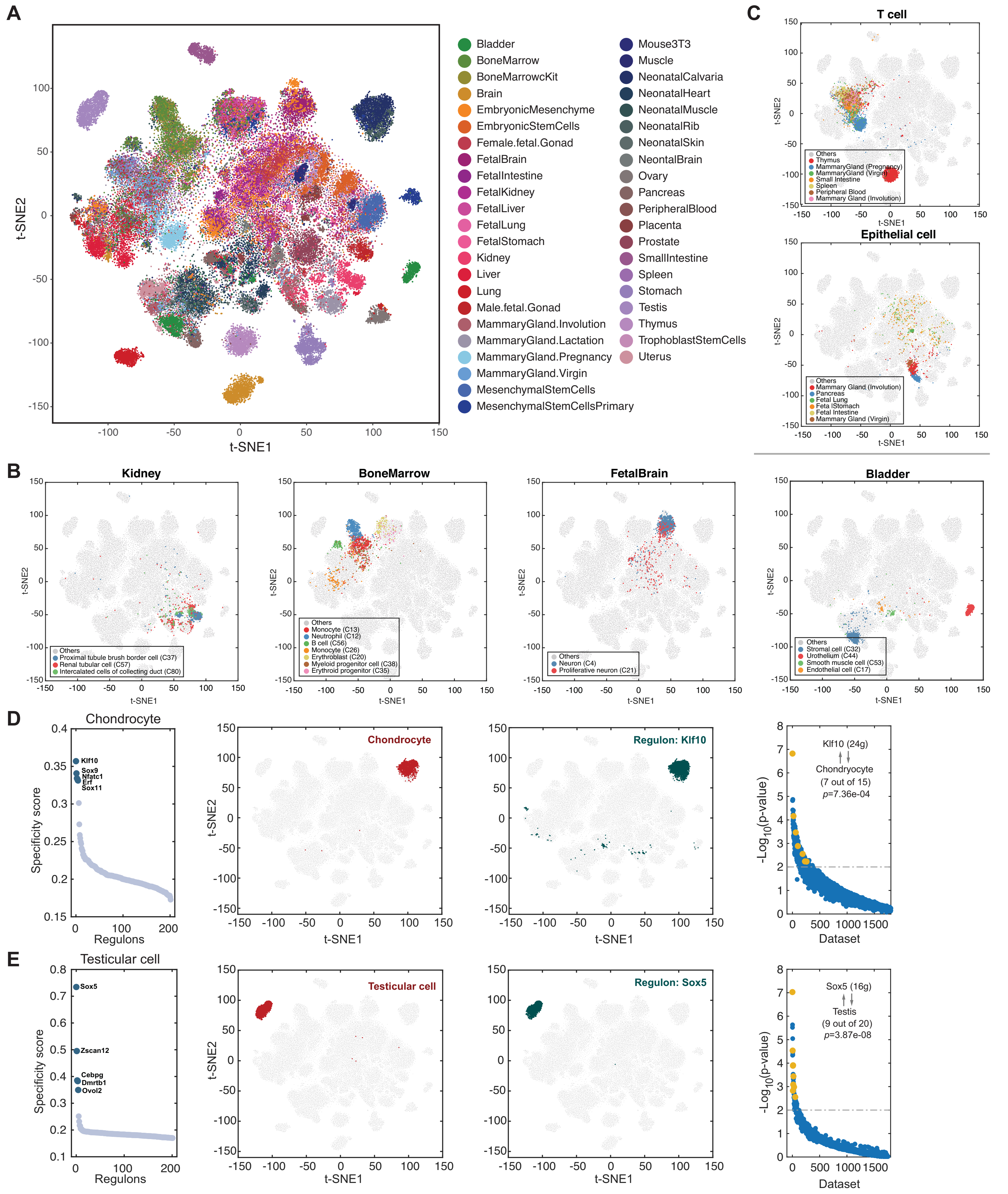

Figure S2. t-SNE map for all sampled single cells (~61k) based on regulon activity score and cell-type specific regulon activity analysis. Related to Figure 1 and Figure 2.

(A) All sampled single cells are highlighted, each cell is color-coded based on tissue. (B) Cells obtained from each individual tissue are highlighted. (C) Cells obtained from each specific cell type are highlighted. (D) Same as Figure 2 but for chondrocyte. (E) Same as Figure 2 but for testicular cell.

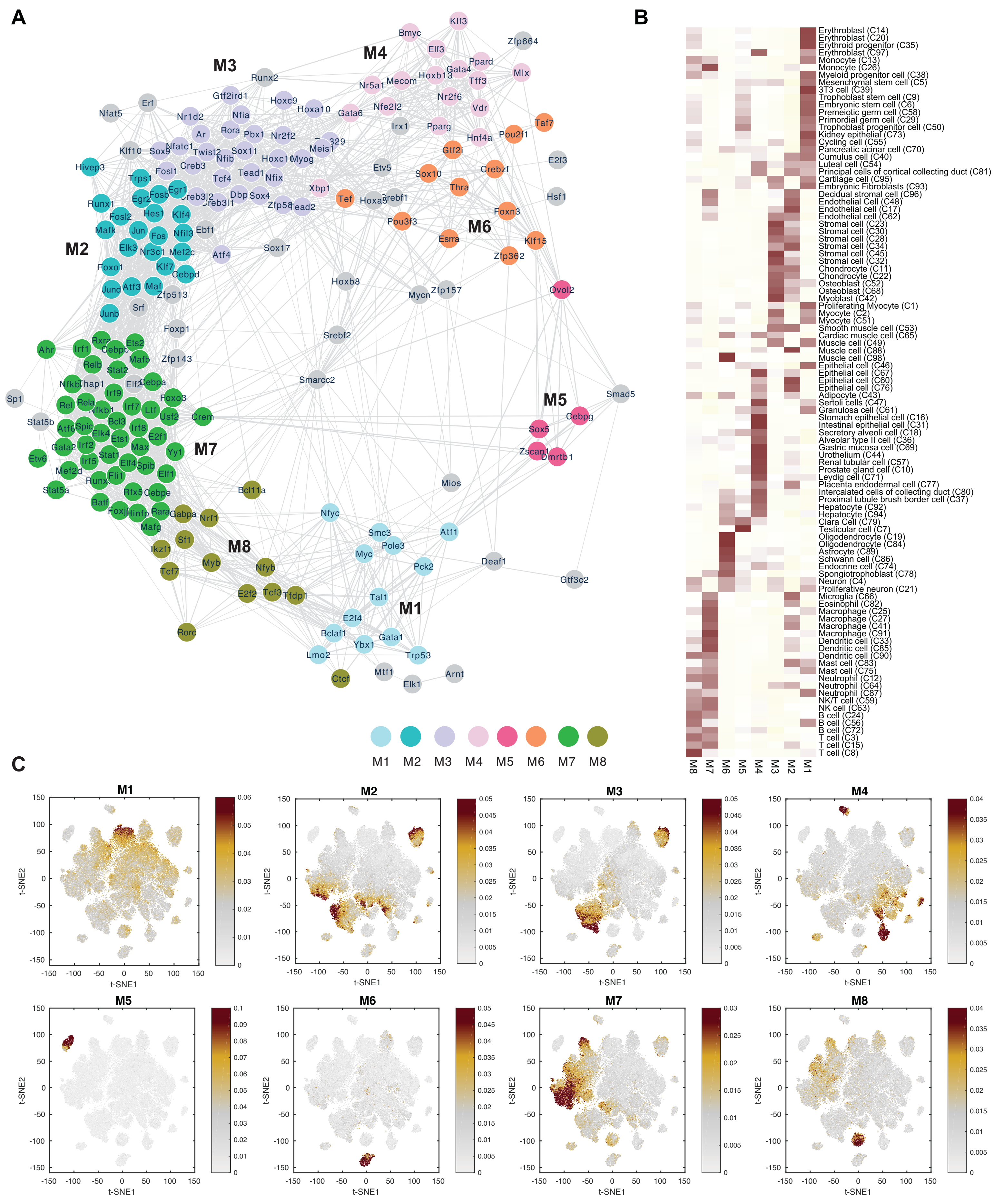

Figure S3. Activation of regulon modules in different cell types. Related to Figure 3.

(A) Regulon association network based on CSI matrix (CSI>0.7). Different colors represent different regulon modules. (B) Average activity scores of 8 representative regulon modules in different cell types. (C) Average module activity scores mapped on t-SNE.

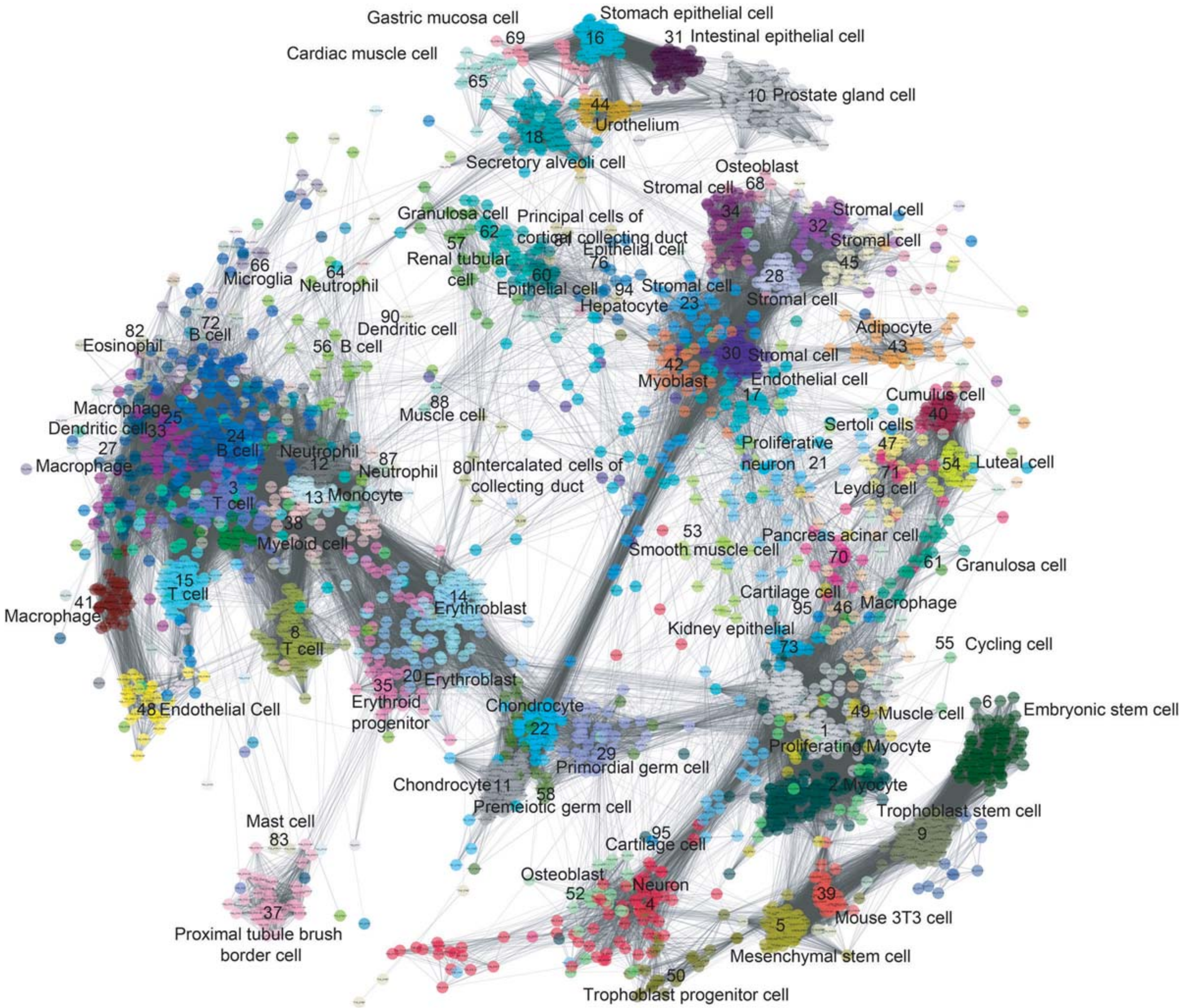

Figure S4. Relationship network for the 98 major cell types. Each node represents a group of 20 cells within the same cell-type and tissue. Related to Figure 4.
